# Use of a cytochrome P450 humanized mouse model to refine schistosomiasis drug discovery

**DOI:** 10.1073/pnas.2600197123

**Published:** 2026-04-10

**Authors:** Sarah D. Davey, Josephine E. Forde-Thomas, Benjamin J. Hulme, Kristin Lees, Alice H. Costain, Mary Evans, Gabriel Rinaldi, Laura Frame, Laste Stojanovski, Frederick R. C. Simeons, Amy Tavendale, A. Kenneth MacLeod, Remi Pichon, Yi-Hsuan Lee, Oktawia Polak, Iain W. Chalmers, Bismark Dankwa, Brenda Kisia Odhiambo, Victor Hugo Guimaraes, Matthew Hegarty, Martin T. Swain, Wayne Aubrey, Nicola Caldwell, Andrew S. MacDonald, Ian H. Gilbert, Beatriz Baragaña, Kevin D. Read, Karl F. Hoffmann

**Affiliations:** ^a^Department of Life Sciences, Aberystwyth University, Wales, Aberystwyth SY23 3DA, United Kingdom; ^b^Lydia Becker Institute of Immunology and Inflammation, Division of Immunology, Immunity to Infection and Respiratory Medicine, School of Biological Sciences, The University of Manchester, England, Manchester M13 9NT, United Kingdom; ^c^Division of Biological Chemistry and Drug Discovery, School of Life Sciences, University of Dundee, Dundee, Scotland DD1 5EH, United Kingdom; ^d^Department of Computer Science, Aberystwyth University, Wales, Aberystwyth SY23 3DA, United Kingdom; ^e^Laboratory of Inflammation and Infectious Diseases, Department of Morphology and Pathology, Federal University of São Carlos, São Carlos CEP 13565-905, Brazil

**Keywords:** *Schistosoma mansoni*, drug discovery, cytochrome P450, humanized mouse

## Abstract

Control of schistosomiasis, a neglected tropical disease caused by infection with *Schistosoma spp*., remains reliant on a single chemotherapy, praziquantel (PZQ). This strategy presents a risk to global health should PZQ-resistant schistosomes establish in endemic areas and justifies the search for new drugs. However, species-specific metabolic differences between humans and preclinical models hinder the optimization of next-generation anti-schistosomal therapeutics. Here, to bypass these species-specific limitations, we exploited a humanized mouse model, 8HUM, engineered to express the principal human Phase I cytochrome P450 enzymes (CYP1A1/2, CYP2C9, CYP2D6, CYP3A4/7) as well as the transcription factors constitutive androstane receptor and pregnane X receptor in place of 35 murine orthologs. We characterized *Schistosoma mansoni* development, immunopathology, hepatic transcriptomic responses, intestinal microbiome changes, and PZQ metabolism as well as PZQ efficacy in 8HUM vs. wild-type (WT) mice. 8HUM mice supported normal *S. mansoni* maturation, infection-associated microbiome dysbiosis, Th2-dominant immune responses, and characteristic hepatic pathology. PZQ intrinsic clearance in 8HUM hepatic microsomes mirrored human levels and was >10-fold lower than that found for WT microsomes. Oral dosing revealed human-like PZQ exposures of (*R*)-PZQ and 4OH-PZQ in 8HUM mice at 25 mg/kg bodyweight and >90% reductions in worm burdens at 100 mg/kg bodyweight (equivalent to that seen in WT mice administered PZQ at 400 mg/kg bodyweight). Our results revealed that 8HUM mice recapitulate key features of murine schistosomiasis while exhibiting human-relevant drug metabolism. These findings establish 8HUM as a refined translational platform for anti-schistosomal drug development, improving predictive accuracy and accelerating therapeutic discovery.

## Significance

Schistosomiasis remains a major global health burden and is treated almost exclusively with praziquantel, despite evidence of emerging resistance and a sparse pipeline of new anti-schistosomals. A key barrier to drug discovery is the poor translational relevance of conventional mouse models, which metabolize drugs very differently from humans. We demonstrate that an advanced genetically humanized mouse model (8HUM) supports normal *Schistosoma mansoni* infection and infection-associated immunopathology while reproducing human-like drug metabolism. This model accurately predicts praziquantel pharmacokinetics and markedly improves in vivo efficacy at clinically relevant exposures. By eliminating misleading, mouse-specific metabolic effects, the 8HUM model enables earlier and more reliable decision-making in anti-schistosomal drug development, with potential to accelerate the delivery of urgently needed new treatments.

Human schistosomiasis is a neglected tropical disease primarily caused by infection with three dioecious blood fluke species (*Schistosoma mansoni*, *Schistosoma haematobium*, and *Schistosoma japonicum*). The disease mainly occurs in tropical and subtropical areas, kills thousands of individuals every year and contributes to an annual loss of up to 4.5 million disability adjusted life years (DALYs) in affected communities (1).

Over the last several decades, schistosomiasis has predominantly been controlled by praziquantel (PZQ), a drug that has recently been shown to activate both a transient receptor potential calcium channel in the parasite (SmTRPM_PZQ_) (2, 3) and a G-protein–coupled receptor in the mammalian host (5HT_2B_) (4). While PZQ is relatively safe and inexpensive to administer, it does have documented limitations (5). One particular limitation includes significant first-pass metabolism in the liver leading to low systemic exposure in the bloodstream (6), which may be partially responsible for lack of in vivo efficacy against juvenile (14 to 28 d old) parasites (7) and ultimately low cure rates in high-transmission areas (8). With preventative chemotherapy programs distributing PZQ at an all-time high, there is also an increased fear that PZQ tolerant or resistant parasites could develop in endemic communities (9, 10). Collectively, these shortcomings support a rationale for identifying new anti-schistosomal compounds with mechanisms of action distinct from PZQ as a component of an integrated strategy to help advance the World Health Organisation goal of schistosomiasis elimination as a public health problem (11).

A critical component of the schistosomiasis drug discovery pipeline is the use of preclinical animal models (e.g., mice) although, unavoidably, there are often major species differences in the metabolic routes of drug elimination. Metabolism of most approved small molecule therapeutics in humans is catalyzed by the cytochrome P450 (CYP) superfamily (12–15). From the four CYP subfamilies (CYP1A, CYP2C, CYP2D, and CYP3A) involved in human xenobiotic metabolism, five produce enzymes responsible for greater than 90% of CYP-mediated drug metabolism (i.e., Phase I metabolism). In contrast, due to both environmental and evolutionary drivers, mice utilize a greater number of genes to catalyze similar Phase I processes (16). Therefore, there are inevitable differences in how rapidly these two species metabolize compounds/drugs. With mice being the dominant preclinical model for testing the in vivo efficacies of antischistosomal compounds, it is likely that mouse-specific metabolic processes have led to the attrition of many promising candidates.

To circumvent this challenge, an extensively humanized mouse line (8HUM) has recently been generated to reflect human Phase 1 drug metabolism pathways (17). This mouse line has had its 33 cytochrome *p450*s of the *cyp1a*, *cyp2c*, *cyp2d,* and *cyp3a* gene subfamilies, together with the transcription factors *car* (*constitutive androstane receptor*) and *pxr* (*pregnane X receptor*) deleted and replaced with the major human Phase I drug metabolizing cytochrome P450 genes *CYP1A1, CYP1A2, CYP2C9, CYP2D6, CYP3A4,* and *CYP3A7*, together with the transcription factors *CAR* and *PXR*. Recently, this model was successfully used to bypass mouse-specific metabolic limitations in efficacy models of *Mycobacterium tuberculosis*, *Leishmania donovani,* and *Trypanosoma cruzi* infection (18), but has yet to be employed in the context of helminth infection.

By demonstrating in this study that 8HUM mice can fully support the sexual development of *S*. *mansoni*, are capable of mounting T helper cell type 2 (Th2)—dominant immunological and pathological responses to infection once egg laying occurs and can be used to quantify metabolism as well as drug-induced efficacy of PZQ, we contend that this model is well-positioned to drive a step-change advance in the schistosomiasis drug discovery pipeline. The use of this translational tool, to complement more traditional approaches in drug discovery, will circumvent mouse-specific metabolism disadvantages and allow optimization of human pharmacokinetics (PK). This refinement has the potential to significantly reduce timelines and associated costs of progressing urgently needed, new antischistosomal candidates.

## Results

### 8HUM Mice Support the Development and Sexual Maturation of *S. mansoni*.

**Fig. 1.**
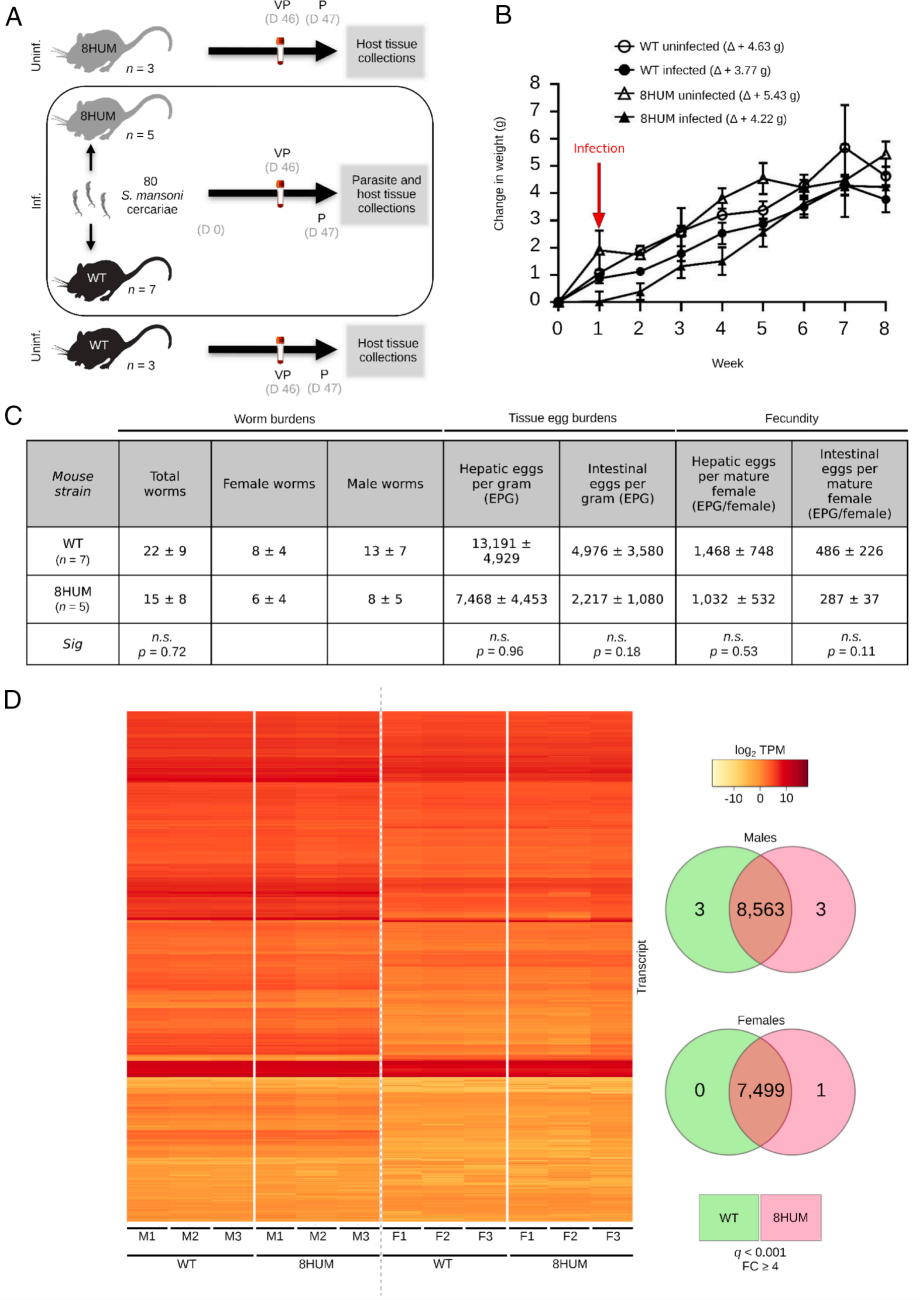
8HUM mice fully support the sexual development of *S*. *mansoni.* (*A*) Schematic overview of experimental process (VP: venous puncture, P: perfusion, Inf.: infected, Uninf: uninfected, D: day). (*B*) Average change in mouse weight from 0 to 8 wk. The change in weight was expressed as the mean weight ± SEM at each time point subtracted from the initial weight at week 0. (*C*) Summary of worm counts, tissue egg burdens, and fecundity. (*D*) Transcript expression (log_2_ transcripts per million, TPM) in male and female worms perfused from WT or 8HUM mice. VENN diagrams to the right of the heatmap represent differential expression at *q* < 0.001 and FC ≥ 4, with shared (non-DE) genes in the overlapping sections (8,563 genes in males and 7,499 genes in females) and significantly up-regulated genes for each strain in non-overlapping sections.

An important prerequisite in the use of 8HUM mice as a refined preclinical replacement to wildtype (WT) mice for early-stage schistosome drug discovery is the knowledge that these animals can support parasite development and mount Th2-dominant immunological and pathological responses to infection at egg-laying onset. To assess these parameters, 80 cercariae were used to initiate percutaneous infection; at days 46 to 47 postinfection, parasitological, and host immunopathological outputs as well as fecal microbiota compositions were compared between 8HUM and WT mouse strains (Fig. 1*A*). Infection did not negatively impact cumulative average weight gain when compared to uninfected controls; in fact, all mice (regardless of genotype or infection status) gained an average weight of ~4 to 5 g (no significant differences between groups; *P* > 0.15) during the 47-d under study (Fig. 1*B*). While 8HUM mice harbored slightly fewer adult male and female schistosomes at the termination of the experiment (47 d postinfection), these worm burden differences were not significant (Fig. 1*C*, *P* = 0.72). To determine if mouse genotype affected total egg burdens or estimates of in vivo schistosome fecundity, eggs laid by adult females were isolated and counted from both liver and intestinal tissues of infected 8HUM and WT mice. Again, no significant differences were observed in the total amount of eggs per gram (EPG) of tissue (*P* = 0.96 for hepatic EPG; *P* = 0.18 for intestinal EPG) or when normalized by numbers of recovered females (*P* = 0.53 for hepatic comparison; *P* = 0.11 for intestinal comparisons; Fig. 1*C*). Furthermore, the sex-ratio normally seen in *S. mansoni* infected mice was also observed in the 8HUM mice (8HUM mice, male: female ratio = 1.3; WT mice, male: female ratio = 1.6) (19).

Previous studies have shown that liver microsomes obtained from 8HUM mice metabolize drugs quite differently than liver microsomes derived from WT mice (18). Therefore, differential liver metabolism in infected 8HUM animals could lead to the production of metabolites that impact the development of adult worms residing in the hepatic portal system. To indirectly assess this hypothesis, we analyzed the transcriptomes of recovered worms by bulk RNA-Seq (Fig. 1*D*). Here, adult male and female transcriptomes were compared from schistosomes obtained from both 8HUM (n = 3) and WT (n = 3) mice using stringent *q* value thresholds (*q* < 0.001) for identifying differentially expressed (DE) transcripts (FC ≥ 4 or log_2_FC ≥ 2) (Fig. 1*D* and Dataset S1). From 9,890 predicted *S. mansoni* protein coding genes (*smps*) present in the *S*. *mansoni* genome assembly (v.10) (Fig. 1*D*, heatmap), a total of 8,563 *smps* were expressed (i.e., transcripts with any detectable expression > 0) in males regardless of the mouse strain in which parasites developed. Only six *smps* were DE (three up-regulated in males derived from 8HUM mice and three up-regulated in males derived from WT mice; Fig. 1*D*, Venn diagram and Dataset S2). Indeed, male transcriptomes were remarkably similar (Pearson correlations presented in *SI Appendix*, Fig. S1). This trend was mirrored in female schistosomes where 7,499 *smps* were expressed (i.e., transcripts with any detectable expression > 0) and only one *smp* was DE in females derived from 8HUM mice (Dataset S2 and Fig. 1*D*, Venn diagram). Even when *q* value thresholds were relaxed (*q* < 0.05), only one additional *smp* was identified as being DE in male transcriptomes (*smp_093430.2;* highlighted row, Dataset S2); no further DE *smps* were identified in female transcriptomes (Dataset S2). These parasitological and transcriptomic data indicate that extensive humanization of the cytochrome P450 pathway did not significantly impact *S. mansoni* development.

### Immunological Responses in 8HUM Mice Are Comparable to WT Animals.

**Fig. 2.**
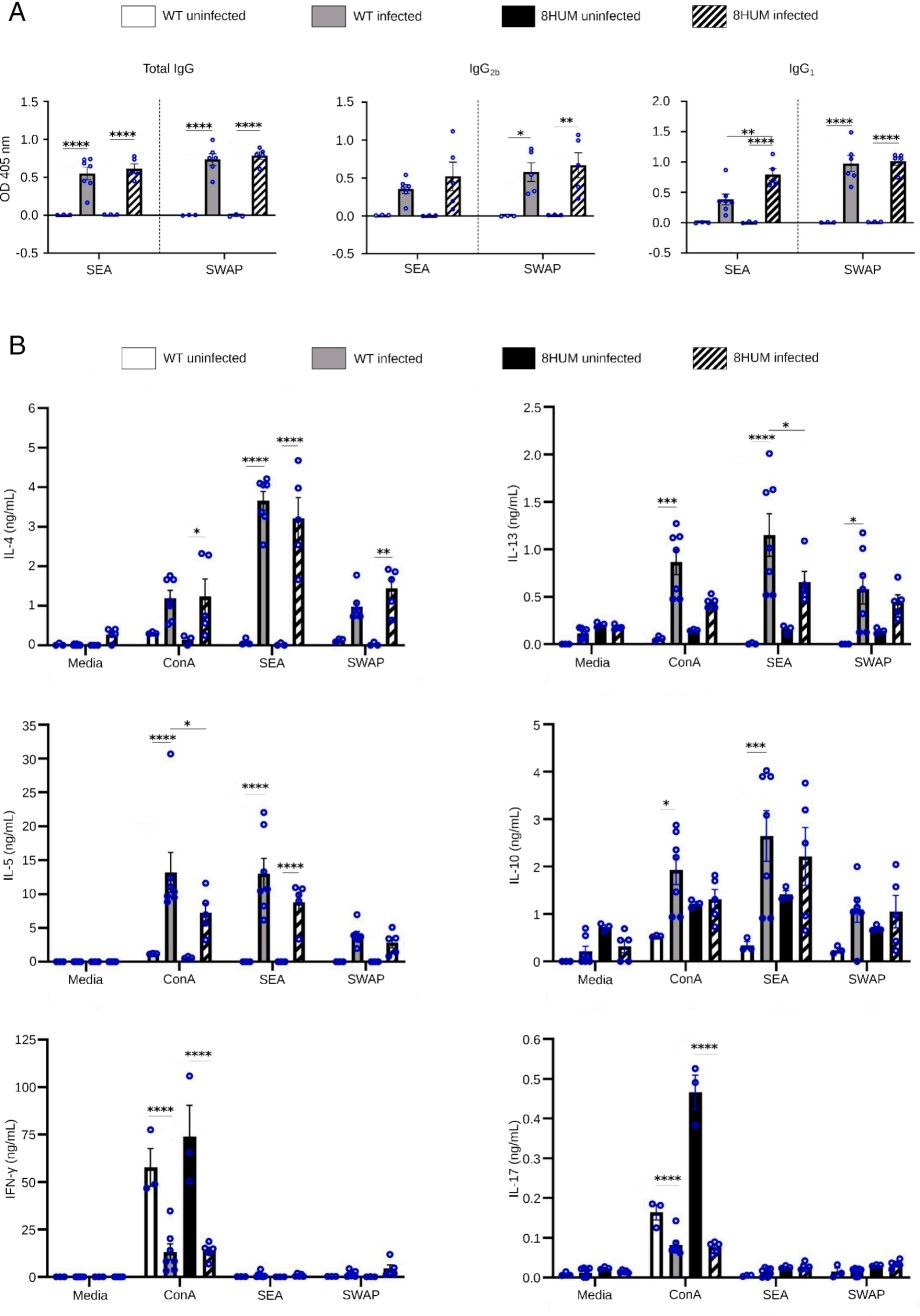
Immunological responses in 8HUM mice are comparable to WT mice. (*A*) Antigen specific IgG antibody responses (Day 46) from mouse sera and (*B*) antigen specific/ConA stimulated cytokine responses of splenocyte cultures (Day 47) were assessed by ELISA. For both, data are expressed as mean ± SEM. Statistically significant differences were calculated using a two-way ANOVA with Tukey’s Multiple Comparison Test. Differences within mouse species and between the two infected strains are shown with an asterisk where *P < 0.05, **P < 0.01, ***P < 0.001, and ****P < 0.0001 (*n* = 3 for WT uninfected and 8HUM uninfected, *n* = 7 for WT infected and *n* = 5 for 8HUM infected). Samples have been background corrected.

Having established that 8HUM mice are susceptible hosts for *S. mansoni*, we next assessed whether they immunologically responded to infection like WT mice (Fig. 2). Serum, obtained from blood samples at day 46 postinfection, demonstrated antisoluble worm antigen preparation (SWAP) IgG (total), IgG_2b_, and IgG_1_ responses that were slightly higher (not significant; *P* > 0.90 for all isotypes) in 8HUM mice when compared to WT animals (Fig. 2*A*). Antisoluble egg antigen (SEA) IgG (total) and IgG_2b_ responses were also slightly higher in infected 8HUM mice compared to infected WT animals (again not significant; *P* = 0.87 and *P* = 0.71 respectively). However, infected 8HUM mice mounted an approximate twofold increase in anti-IgG_1_ SEA that was statistically different from that measured in infected WT animals (*P* = 0.01).

To compare antischistosomal cellular responses to infection, splenocytes derived from 8HUM and WT spleens at day 47 postinfection were restimulated with SWAP and SEA (Fig. 2*B*). Th1 (IFN-gamma), Th2 (IL-4, IL-5, and IL-13), anti-inflammatory (IL-10) and Th17 (IL-17) cytokines were measured in the culture supernatants at 72 h poststimulation. SWAP and SEA-specific splenic production of Th2 cytokines (IL-4, IL-5, and IL-13) and anti-inflammatory IL-10 were all elevated upon schistosome infection in both 8HUM and WT mice. In contrast, very little IL-17 or IFN-gamma was produced in response to antigen (or Concanavalin A; ConA) stimulation in infected 8HUM or WT splenocyte cultures despite both cytokines being detectable in uninfected splenocyte supernatants stimulated by ConA. SEA induced IL-13 (as well as ConA stimulated IL-5) was the only antigen-specific cytokine that was statistically reduced in 8HUM splenocyte cultures when compared to WT samples (*P* = 0.02). Taking both antigen-specific antibody and splenic cytokine responses together, these data suggest that, although there were some differences in baseline levels, 8HUM mice responded to *S. mansoni* infection similarly to WT mice.

### Infection-Associated Fecal Microbiota Dysbiosis Occurs in 8HUM Mice.

*S. mansoni* infection in WT mice affects host microbiota dysbiosis (i.e., reduced alpha diversity) in fecal matter collected from intestines (20, 21). To understand if this infection-associated process develops similarly in 8HUM mice, 16S rRNA sequencing of microbial DNA isolated from intestinal contents was compared between 8HUM and WT mouse groups (uninfected and 47 d postinfection; *SI Appendix*, Fig. S2). As previously reported (20, 21), *S. mansoni* infection of WT mice led to a decrease in alpha diversity of fecal microbiota communities (*SI Appendix*, Fig. S2*A*). A similar trend was also observed in 8HUM fecal samples, indicating that infection of both mouse strains was linked to a reduction in bacterial species richness in intestinal contents. However, differences in species composition (i.e., beta diversity) were predominantly driven by mouse genotype, regardless of infection status (*SI Appendix*, Fig. S2*B*). Collectively, these observations are entirely consistent with previous reports studying schistosome-associated effects on microbiota in different mouse strains (20, 21) and suggest that extensive humanization of the cytochrome P450 pathway is not a confounder.

### Infection-Induced Hepatic Transcriptome Profiles Are Minimally Affected by Cytochrome P450 Humanization.

**Fig. 3.**
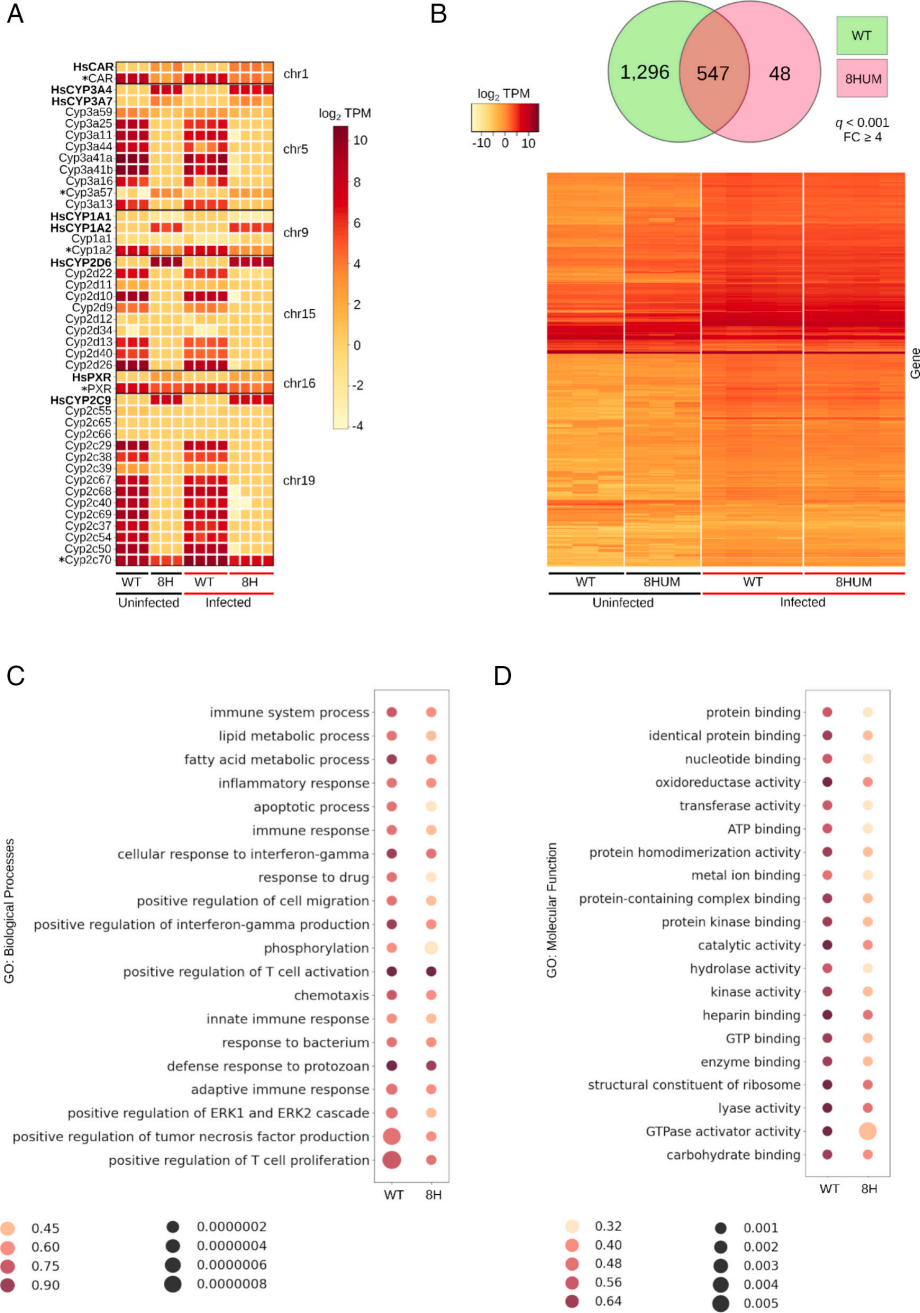
Hepatic transcriptomes in 8HUM mice respond to *S. mansoni* infection similarly to WT mice. (*A*) Expression heatmaps (log_2_ transcripts per million, TPM) of the 35 *cytochrome p450* genes and transcription factors *car* and *pxr* substituted for human orthologs in 8HUM mice (*cyp3a41* has two paralogs: *cyp3a41a* and *cyp3a41b*) in addition to their 8 humanized counterparts. * indicates mouse genes with residual transcription in 8HUM (max TPM >10). (*B*) Expression (log_2_ TPM) of DE genes (*q* < 0.001 and FC ≥ 4) between uninfected and infected (at D47 postinfection) WT and 8HUM hepatic transcriptomes. The VENN diagram above the heatmap indicates distinct and shared genes which are DE in response to infection. (*C*) Top 20 enriched GO terms for gene sets within Biological Processes and (*D*) Molecular Function in response to infection. In both bubbleplots (*C* and *D*), enrichment ratio (proportion of genes annotated by a term that are significantly enriched where 1 = all genes enriched) is shown by dot color and significance is shown by dot size (smaller dots = more significant, smaller *q* value).

*S. mansoni* infection in WT mice leads to transcriptional changes in hepatic genes responsible for inflammation, extracellular matrix remodeling, and wound healing (22, 23). To quantify how these pathology-associated features develop in 8HUM mice, bulk RNA-Seq of liver tissues was compared between WT and 8HUM animals (uninfected and 47 d postinfection groups; Fig. 3). As the 8HUM mice should be deficient in 35 transcripts derived from four *cyp450* gene subfamilies (*cyp1a*, *cyp2c*, *cyp2d, and cyp3a*) as well as the transcription factor *car* and *pxr* (17), we first examined liver transcriptomes from highly correlated replicates (n = 3 for uninfected samples, n = 4 for infected samples; *SI Appendix*, Fig. S3) for evidence of these products’ expression (Fig. 3*A* and Dataset S3). Unsurprisingly, and regardless of infection status, 8HUM transcriptomes had negligible expression (average TPM ≤ 2) for most mouse *cyp450* subfamilies (including two *cyp3a41* paralogs, *cyp3a41a* and *cyp3a41b*). Five genes (*car*, *cyp1a2*, *cyp2c70*, *cyp3a57*, and *pxr*) were still detectable (maximum TPM > 10) in 8HUM hepatic transcriptomes, however, all cases were explained by residual mappings to incomplete transcripts (e.g., retention of non-functional exons 6 to 9 in *cyp2c70*) or UTR sequence (*SI Appendix*, Fig. S4). In WT mice, schistosome infection led to a reduction in most members of these cytochrome *p450* gene subfamilies and transcription factors; *cyp2c70* was the single exception and was significantly induced upon infection (average TPM counts = 488.24 in uninfected animals; average TPM counts = 3,759.23 in infected animals, *q* < 0.001, log_2_FC < 0.530).

Expression of knocked-in human genes was also detected in all 8HUM samples, with *CYP2C9* most highly expressed (average TPM 695.46 for uninfected mice and 398.68 for infected mice, Dataset S3). Most 8HUM humanized cytochromes decreased in expression in response to infection, although this decrease was only significant (*q* < 0.05) in two instances: *CYP2C9* (*q* < 0.01, log_2_FC = 0.451) and *CYP3A4* (*q* < 0.05, log_2_FC = 0.664). Transcription factor *CAR* was the exception, marginally increasing in response to infection (from an average TPM of 8.899 to 14.232); however, this change was not significant (*q* = 0.436, log_2_FC = 0.370).

Next, we examined how schistosome infection/egg embolization globally affected transcription in the liver. From a total of 17,572 *M. musculus* protein coding genes (genome assembly GRCm39) with detectable expression > 0 (Dataset S4), we quantified hepatic transcripts that were DE (up- or down-regulated) upon infection in each mouse strain using a stringent *q* value cutoff of 0.001 and a FC ≥ 4 (Fig. 3*B* and Dataset S5). Using these criteria, we found that infection led to the differential expression of 1,891 genes (heat map, Fig. 3*B*). When deconvoluting these results according to mouse strain, infection in WT mice led to the differential expression of 1,296 unique liver transcripts, whereas infection in 8HUM animals led to the differential expression of 48 unique liver transcripts; an overlapping set of 547 transcripts were DE upon infection in both mouse groups (Venn diagram, Fig. 3*B*). Gene set enrichment analyses (GSEA) revealed a top 20 list of shared Gene Ontology (GO) Biological Processes (BP) and Molecular Function (MF) terms that were significantly over-represented upon infection (*q* < 0.05) in both 8HUM and WT mice (Fig. 3 *C* and *D*, respectively). As illustrated in the bubbleplots, the numbers of enriched genes within the top 20 term sets were higher in response to infection in all BP (Dataset S6) and MF (Dataset S7) terms in WT mice compared to 8HUM mice. This GSEA demonstrated that both mouse strains mounted a qualitatively similar transcriptional response to schistosome infection/egg embolization in the liver, though, quantitatively the proportion of DE genes within each gene set varied between strains.

### Characteristic Hepatic Pathology Develops in Infected 8HUM Mice.

**Fig. 4.**
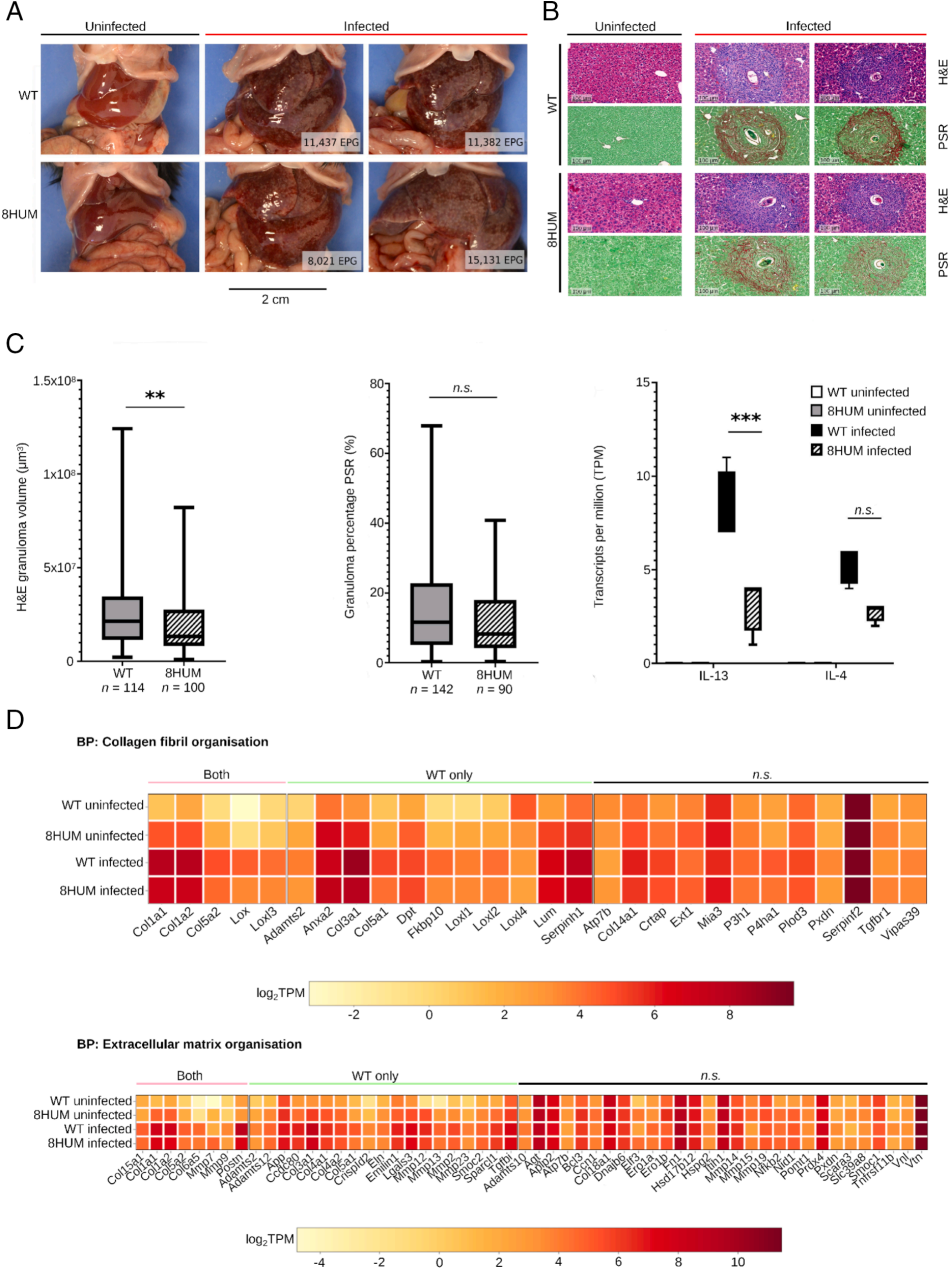
8HUM mice develop characteristic granulomatous hepatic pathology upon *S. mansoni* infection. (*A*) Representative necropsy images from livers of uninfected (*n* = 1) and infected (*n* = 2) WT and 8HUM mice. Average EPGs of liver tissue for representative infected individuals are provided in white boxes. (*B*) Uninfected (*n* = 1) and infected (*n* = 2) mouse livers stained with H&E and PSR. (*C*) Quantification of liver pathology-associated parameters include: granuloma volumes calculated from H&E areas assuming perfect sphericity (*P* < 0.01 according to Mann–Whitney *U* test); percentage collagen content per granuloma as measured by quantification of PSR+ tissue (*P* = 0.09 according to Mann–Whitney *U* test); infection-induced increases in expression of cytokines *il-13* (*q* < 0.001, log_2_FC = 1.39) and *il-4* (*q* = 0.15, log_2_FC = 0.81). (*D*) Expression heatmaps (log_2_ TPM) of genes annotated by GO terms collagen fibril organization (GO:0030199) and extracellular matrix organization (GO:0030198) with > 10 TPM across all replicates. Genes have been categorized into either “Both”, “WT only” or “n.s.” to indicate significant differential expression in both WT and 8HUM, significant differential expression in WT only or no significant differential expression in response to infection, respectively.

*S. mansoni* infection in WT mice leads to the formation of collagen-enriched granulomas around eggs trapped in an inflamed liver (as reviewed in ref. 24). Upon gross pathological examination of murine pathology at necropsy, schistosome infection clearly led to similar, egg-induced macroscopic lesions in enlarged 8HUM livers (Fig. 4*A*). Histological examination of these lesions clearly revealed defined (hematoxylin and eosin; H&E) and collagen-rich (picrosirius red; PSR) granulomas (Fig. 4*B*). However, these hallmarks of murine schistosomiasis were slightly larger (*P* < 0.01) and more collagen-enriched (*P* = 0.09) in WT mice (Fig. 4*C*). The modest increase in granulomatous pathology observed in WT mice correlated with similar increases in liver *il-13* (*q* < 0.001, log_2_FC = 1.39) and *il-4* (*q* = 0.15, log_2_FC = 0.81) (Fig. 4*C*), two cytokine gene products responsible for positively regulating granuloma formation and hepatic fibrosis during murine schistosomiasis (25). Further GO analyses of BP terms involved in fibrosis and granuloma formation [collagen fibril organization (GO:0030199), Dataset S8; and extracellular matrix organization (GO:0030198), Dataset S9] provided additional support for the hepatic pathology differences observed between infected WT and 8HUM mice (Fig. 4*D*). Here, a subset of genes in each BP term was significantly up-regulated in response to infection in both WT and 8HUM animals (Fig. 4*D*, Both); however, a larger subset was significantly increased upon infection in WT mice only (Fig. 4*D*, WT only). Many of these genes have previously been positively linked to granuloma formation and hepatic fibrosis during murine schistosomiasis (22, 23, 26–28); their greater induction in WT mice upon infection provides an explanation for the modest increases in granuloma volume and intragranuloma fibrosis observed in our study. However, despite differences in magnitude, these histopathological and transcriptional findings collectively indicated that 8HUM mice develop the characteristic Th2-driven, pathological features of experimental schistosomiasis seen in WT animals.

### Translational Relevance of PZQ Metabolism Is Improved in the 8HUM Model.

**Fig. 5.**
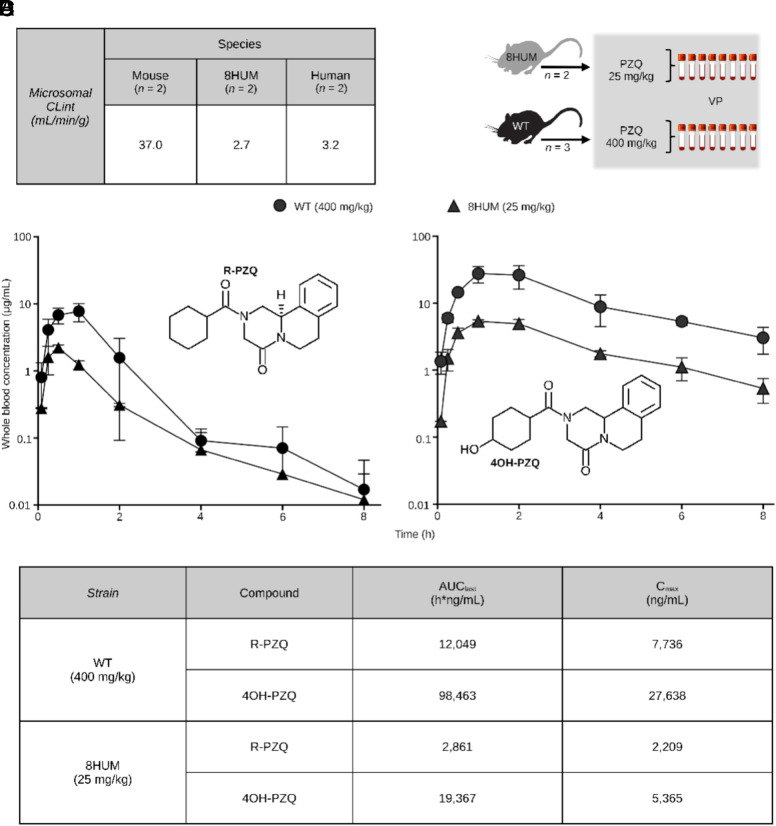
8HUM mice exhibit human-equivalent PZQ metabolism. (*A*) In vitro clearance rate of PZQ by liver microsomes (*n* = 2 for each species; intrinsic clearance: CLint). (*B*) Schematic overview of range-finding pharmacokinetic study. Blood samples were obtained by venepuncture (VP) at 5, 15, 30, 60, 120, 240, 360, and 480 min after rac-PZQ administration. (*C*) Concentration vs. time plots of total blood levels are shown for R-PZQ and 4OH-PZQ for both WT (*n* = 3; 400 mg/kg) and 8HUM (*n* = 2; 25 mg/kg) animals following oral administration of rac-PZQ (mean concentrations ± SD shown). (*D*) Summary PK exposure levels for R-PZQ and 4OH-PZQ in rac-PZQ dosed WT and 8HUM mice.

To investigate the suitability of the 8HUM model for assessing drug-induced efficacy in schistosome-infected animals, we first explored the in vitro clearance rate (intrinsic clearance; CL_int_) of PZQ by 8HUM hepatic microsomes and in vivo PK of PZQ in uninfected 8HUM mice (Fig. 5). In vitro, 8HUM and human hepatic microsomes metabolized racemic PZQ (rac-PZQ) at a similar rate (8HUM CL_int_ = 2.7 mL/min/g, human CL_int_ = 3.2 mL/min/g); noticeably, these CL_int_ values were > 10× lower than that measured for WT mouse hepatic microsomes (CL_int_ = 37.0 mL/min/g) (Fig. 5*A*). In vivo, as measures of exposure (AUC and C_max_) in 8HUM mice can be far higher than in WT mice administered the same dose (18), we conservatively orally dosed 8HUM animals with 25 mg/kg rac-PZQ compared to 400 mg/kg rac-PZQ in WT mice for an initial range-finding PK study (Fig. 5*B*). Both groups of treated animals displayed similar PK profiles for the active enantiomer of PZQ (i.e., *R*-PZQ) and the major metabolite of PZQ (4OH-PZQ) throughout the study (Fig. 5*C*). Despite a 16-fold difference in administered dose, AUC_last_ and C_max_ of *R*-PZQ in 8HUM animals were only ~fourfold lower when compared to WT animals; AUC_last_ and C_max_ for 4OH-PZQ in 8HUM animals were only ~fivefold lower (Fig. 5*D*). Based on these exposure differences, and assuming a linear relationship between dose and exposure, we predicted that oral administration of a single dose of ~100 to 200 mg/kg rac-PZQ to *S. mansoni*–infected 8HUM animals would lead to similar levels of parent drug and metabolite exposure and, thereby, drug-induced efficacies (~94%) comparable to that typically observed in WT animals receiving a single oral dose of 400 mg/kg rac-PZQ (29).

### Validation of the 8HUM Model for Quantifying PZQ-Induced Efficacy against *S. mansoni*.

**Fig. 6.**
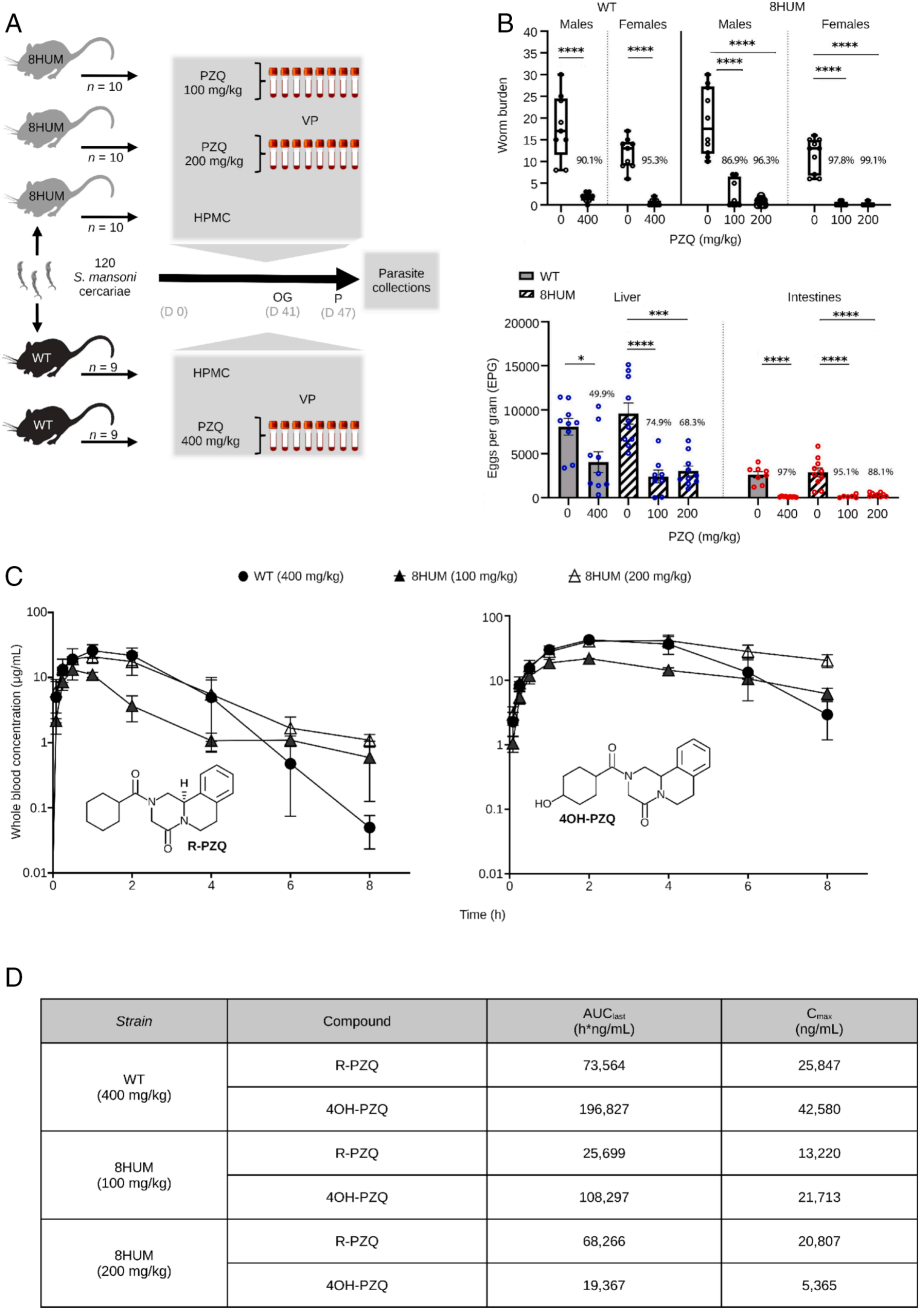
8HUM mice are an improvement over WT animals in preclinical efficacy studies. (*A*) Schematic overview for quantifying PZQ-induced efficacy in *S. mansoni* infected mice [VP: venous puncture, P: perfusion, OG: oral gavage, HPMC: hydroxypropyl methylcellulose (vehicle control), D: day]. (*B*) The percent worm and egg reductions following exposure to PZQ is indicated above each of the groups and was calculated according to the formula: (untreated mean–PZQ treated mean/untreated mean × 100). Statistically significant differences in worm and egg burdens between untreated and PZQ-treated groups were determined by a one-way ANOVA with Tukey’s Multiple Comparison Test where *P < 0.05, ***P < 0.001 and ****P < 0.0001. (*C*) Concentration vs. time plots of total blood levels are shown for R-PZQ and 4OH-PZQ for both WT (*n* = 3, 400 mg/kg) and 8HUM (*n* = 3, 100 mg/kg; *n* = 3, 200 mg/kg) animals following oral administration of rac-PZQ (mean concentrations ± SD shown). (*D*) Summary PK exposure levels for R-PZQ and 4OH-PZQ in rac-PZQ dosed WT and 8HUM mice.

To test this prediction, groups of WT and 8HUM mice were infected with 120 *S. mansoni* cercariae and the efficacy of rac-PZQ treatment in differentially dosed groups was compared (Fig. 6). Specifically, at 41 d postinfection, 8HUM animals were orally dosed with excipient only (0 mg/kg; n = 10), 100 mg/kg (n = 10) or 200 mg/kg (n = 10) rac-PZQ. In comparison, WT animals were orally dosed with excipient only (0 mg/kg; n = 9) or with 400 mg/kg (n = 9) rac-PZQ (Fig. 6*A*). At day 47 postinfection, worm and egg burdens were compared between all groups (Fig. 6*B*). As previously observed (29), a single 400 mg/kg dose of rac-PZQ in WT mice led to a significant reduction (93%) in adult worm burdens [females (95%) > males (90%)] when compared to excipient controls. While a similar worm burden reduction (92%) was observed in 8HUM mice receiving a single 100 mg/kg dose of rac-PZQ, a greater worm burden reduction (98%) was found in the 8HUM group receiving a single 200 mg/kg dose of rac-PZQ (*P* > 0.99). Comparable to treated WT animals, rac-PZQ had a larger effect on female (vs. male) worm burdens in both 8HUM-treated groups. The effect on liver and intestinal egg burdens broadly mirrored worm burden differences found among all rac-PZQ treated groups.

PK analyses of blood samples derived from rac-PZQ-treated, infected animals demonstrated a “flattening out” effect for both *R*-PZQ and 4OH-PZQ curves in the 8HUM groups compared to the WT group (Fig. 6*C*). This phenotype has been observed before in 8HUM animals dosed with a variety of structurally diverse, anti-infective medicines and may be driven by slower drug/compound elimination (18). Exposure (i.e., AUC_last_ and Cmax values) of *R*-PZQ and 4OH-PZQ in infected 8HUM animals tracked alongside dosage administered and, for the 200 mg/kg treatment group, approached or exceeded the levels observed in infected WT mice dosed with 400 mg/kg (Fig. 6*D*). These results support the accuracy of our estimated rac-PZQ dosing range (100 to 200 mg/kg) required to elicit worm and egg burden reductions in infected 8HUM mice comparable to those observed in infected WT mice administered 400 mg/kg rac-PZQ.

## Discussion

Schistosomiasis is a neglected tropical disease predominantly managed by monotherapy with PZQ. Considering that there are no vaccines near clinical use (30), there are potential PZQ-resistant schistosome populations already established in high transmission areas of East Africa (10) and there is only one advanced PZQ replacement candidate under investigation as a late lead (31), the development of new treatments for schistosomiasis remains a public-health priority. To accelerate the progression of actionable new anti-schistosomal therapeutics, we comprehensively detail how the most advanced genetically humanized mouse model for studying Phase I metabolism [8HUM (17)] can be successfully used as an integral component of current schistosome drug discovery pipelines.

As 8HUM mice have not previously been tested for their susceptibility to *S. mansoni* infection, nor have they been formally assessed for PZQ exposure during PK investigations, we sequentially conducted our study in three distinct phases to mitigate any adverse events. The first phase involved a robust characterization of the salient features of schistosome infection in this mouse model and quantitatively assessed parasite development (Fig. 1), host responses to infection (Figs. 2–4) and intestinal microbiota dysbiosis (*SI Appendix*, Fig. S2). When contrasted to infection in WT mice, most outputs measured in infected 8HUM mice were comparable. Nevertheless, slight differences in hepatic pathology (granuloma volume, intra-granuloma fibrosis and genes associated with both collagen fibril- and extracellular matrix- organization) were observed (Fig. 4). While not always significant, these differences were correlated with IL-13 levels (splenic-derived protein: Fig. 2, liver-derived transcript: Fig. 4). As parasite-induced IL-13 drives hepatic pathology during murine schistosomiasis (25), the slight decrease in parasitological load found in infected 8HUM mice (Fig. 1) could, in part, explain these liver alterations. However, metabolites differentially created in response to the cytochrome P450 repertoire of infected 8HUM animals, could also contribute to these observations. In support of this contention, a recent study concluded that 8HUM hepatic microsomes incubated with 14 approved human medicines created products that are more comparable to human than WT mouse metabolites (18). Therefore, global metabolomic profiling, in response to *S. mansoni* infection, is currently being performed in 8HUM mice. These metabolites might also contribute to the slight, but significant, differences observed in adult schistosome transcriptomes derived from 8HUM mice (six DE transcripts in adult males and one in adult females; Fig. 1*D* and Dataset S2). Nevertheless, our comprehensive characterization of *S. mansoni* development and schistosomiasis-related changes in intestinal microbiota, immunology, and hepatic immuno-pathology clearly indicates that 8HUM mice are suitable replacements for WT mice in the schistosome drug discovery pipeline.

The second phase of our studies involved the in vivo (PK) and in vitro (CL_int_) characterization of rac-PZQ metabolism in uninfected 8HUM mice (Fig. 5). The finding that 25 mg/kg bodyweight rac-PZQ administered to 8HUM mice led to systemic exposures of *R*-PZQ (AUC_last_ = 2,861 h*ng/mL) similar to those found in humans given 20 to 40 mg/kg bodyweight rac-PZQ [AUC_last_ = 1,303 to 4,830 h*ng/mL; (32, 33)] clearly indicates comparable rates of drug metabolism. 4OH-PZQ (both *R* and *S* enantiomers) systemic exposures in 8HUM mice (AUC_last_ = 19,367 h*ng/mL) also was within the range found for (*R*)-4OH-PZQ in human (children) populations given 20 to 40 mg/kg bodyweight rac-PZQ (AUC_last_ = 16,840 to 56,590 h*ng/mL) (summarized in ref. 34). Similarly, both 8HUM- and human- hepatic microsomes cleared rac-PZQ at near identical rates (CL_int_ values 2.7 mL/min/g vs. 3.2 mL/min/g, respectively), which was substantially lower than that measured for WT- hepatic microsomes (CL_int_ = 37 mL/min/g). Thus, the removal of high activity mouse enzymes that limit exposure to PZQ highlights a key advantage of using 8HUM mice in early-stage drug efficacy studies of schistosomiasis. This strategy enables the prediction of pharmacokinetic exposures of medicines and late lead compounds that more closely reflect human metabolism, thereby allowing earlier and better-informed go/no-go decisions and minimizing unnecessary medicinal chemistry optimization driven by mouse-specific metabolic products. Future studies should address whether and to what extent the use of 8HUM data may improve the accuracy of human dose prediction, both in relation to efficacy and toxicity.

The third phase of our investigation aimed to validate the 8HUM model as an improvement over WT mice in assessing the efficacy of PZQ against *S. mansoni*. In rac-PZQ dosed WT mice, *R*-PZQ and 4OH-PZQ exposures were higher in infected animals (compare Fig. 6*D* vs. Fig. 5*D*); this difference is correlated with decreased hepatic expression of *cytochrome p450*s (Fig. 3*A*) and has been observed before (35). While we have not performed similar comparisons in 8HUM animals (uninfected vs. infected, treated with the same dose of rac-PZQ), it is possible that the decreased expression of the “humanized” *cytochrome p450*s in response to infection (Fig. 3*A* and Dataset S3) may be responsible for the leveling out of *R*-PZQ and 4OH-PZQ exposures observed in infected 8HUM animals (Fig. 6*C*). Nevertheless, rac-PZQ is metabolized extremely rapidly in WT mice [Fig. 5 and (36)], necessitating a single high oral dose of 400 mg/kg body weight to sustain systemic—and likely mesenteric—exposures of *R*-PZQ sufficient to achieve substantial worm burden reductions (>90%) (29). Optimal PZQ-mediated efficacy in WT mice also depends on an intact immune system (37, 38). Although 8HUM mice exhibit immune responses to *S. mansoni* infection comparable to those of WT mice (Fig. 2), their humanized cytochrome P450 profile resulted in markedly enhanced rac-PZQ efficacy, achieving >90% reductions in worm burdens (>70% reductions in egg burdens) at single oral doses of 100 to 200 mg/kg bodyweight; these parasitological reductions exceeded (tissue eggs) or were comparable (worms) to the efficacy observed in WT mice dosed with 400 mg/kg bodyweight (Fig. 6). Notably, while a single 100 mg/kg bodyweight oral dose of rac-PZQ produces (at most) a 53% reduction in worm burden in WT mice (7), the approximately twofold increase in efficacy observed in 8HUM mice at the same dose underscores the translational advantage conferred by humanized drug metabolism in this model.

The 8HUM model incorporates the major human cytochrome P450 enzymes responsible for most CYP-mediated drug metabolism. However, it notably lacks CYP2C19 and CYP2J2. These isoforms, together with CYP1A2, CYP2C9, CYP2D6, and CYP3A4 (all of which are present in 8HUM mice), contribute substantially to rac-PZQ metabolism in humans (39–41). Consequently, direct extrapolation of findings related to Phase I metabolism from the 8HUM model to humans should be made with caution especially when also considering pharmacogenetic variations responsible for PZQ metabolism in endemic populations (42). Moreover, 8HUM mice retain several murine CYP subfamilies (e.g., Cyp2a, Cyp2b, and Cyp2e), whose residual activity—alongside that of Phase II conjugating enzymes and Phase III transporters—may influence the metabolic fate of novel compounds. As we have previously shown, compounds for which metabolic elimination in 8HUM is predominantly mediated by the retained murine Cyps can be identified through a counter screen using hepatic microsomes from the CypC4KO mouse line (18). In this line, all Cyps in the Cyp1a, 2c, 2d, and 3a gene families have been deleted from the mouse genome but no human CYPs have been introduced. Consistent with observations in humans (14), the proportion of compounds falling into this category is less than 20% of approved medications. Nonetheless, further humanization of these CYP isoforms and Phase II/III proteins could enhance the translational fidelity of the 8HUM model for schistosomiasis drug discovery.

In summary, this study demonstrates that replacing WT mice with 8HUM animals in schistosome drug discovery pipelines effectively eliminates species-specific differences in drug metabolism, thereby aligning compound optimization more closely with human PK. By enhancing translational relevance and improving the efficiency of development through reduced time and cost, 8HUM mice represent a powerful and practical refinement to WT mice, with the potential to transform preclinical evaluation and accelerate the discovery of human therapeutics for schistosomiasis.

## Materials and Methods

### Experimental Design.

This study was designed to establish the 8HUM mouse model as a translational tool for assessing the in vivo efficacy of antischistosomal compounds during preclinical stages of drug discovery. The project had three phases. The first phase evaluated the 8HUM model as a suitable replacement for WT mice in supporting the sexual maturation of *S. mansoni* parasites and in permitting the development of infection-related characteristics. The second phase quantified the in vitro (intrinsic microsomal clearance, CL_int_) and in vivo (pharmacokinetics, PK) metabolism of racemic-praziquantel (rac-PZQ) in the 8HUM model. Using information derived from the PK studies, the third phase evaluated the efficacy of rac-PZQ in *S. mansoni* infected 8HUM mice at exposure levels far lower than that required to induce high levels (>90%) of efficacy in WT mice. Experimental design was guided by power calculations (G*Power; https://www.gpower.hhu.de), previous experience, and pilot studies. All animal work was undertaken in line with the 3Rs principles of replacement, reduction, and refinement (https://www.nc3rs.org.uk).

### Ethics.

All regulated procedures described in this study were presented to and approved by the Animal Welfare and Ethical Review Bodies (AWERB) at Aberystwyth University (AU) and University of Dundee (UoD). They adhered to the United Kingdom Home Office Animals (Scientific Procedures) Act of 1986 and were performed according to project licenses PP2955700 (AU) and PP5016780 (UoD). Animals were inspected regularly by staff trained and experienced in small animal husbandry, with 24 h access to veterinary advice. Mice were maintained with ad libitum access to food and water, and a 12-h light/dark period. Temperature and relative humidity were maintained between 20 °C and 24 °C, and 45% and 65%, respectively.

### Chemicals and Reagents.

Racemic-PZQ (rac-PZQ) for administration in PK studies was purchased from Sigma Aldrich/Merck (Burlington, MA). (*R*)-PZQ, (*S*)-PZQ and 4OH-PZQ for bioanalysis were purchased from Stratech (Ely, UK), CliniSciences (Nanterre, France), and Accela ChemBio (San Diego, CA), respectively. All LC–MS/MS mobile phase reagents were purchased from Fisher Scientific (Thermo Fisher Scientific, Waltham, MA).

### *S. mansoni* Lifecycle Maintenance.

For *S. mansoni* (Naval Medical Research Institute; NMRI strain) lifecycle maintenance, 6- to 26-wk-old female TO (HsdOLa:TO - Tuck Ordinary; Envigo, UK) mice were percutaneously exposed by immersion of their tails in water containing 180 cercariae for 45 min. At 47 d postinfection, animals were administered an intraperitoneal injection of a lethal dose of sodium pentobarbital containing 100 U/mL heparin and parasites collected by reverse perfusion of the hepatic-portal venous system. Following perfusion, eggs were recovered from infected livers, miracidia hatched and used to infect susceptible *Biomphalaria glabrata* snails [NMRI albino and pigmented outbred strains (43)].

### Phase 1 Experimental Infections.

All mice included in the study were housed in Aberystwyth University’s biological research facility for 3 wk before investigations began. For experimental infection of transgenic 8HUM (17) (C57BL6/NTac background; PhaSER Biomedical, UK) and WT C57BL6/NCrl (Charles River Laboratories, UK) mice, 6- to 8-wk-old females (5 to 7 animals per group) were percutaneously exposed as above to 80 cercariae/mouse. Groups of uninfected animals (three animals per group; 8HUM and WT) were housed alongside their infected counterparts. All mice in the study were weighed individually, prior to, and weekly after experimental infection. At 46 d postinfection, blood samples (~100 µL) were taken from the lateral tail veins of individual mice (infected and uninfected controls) for serum preparation. At 47 d postinfection, animals were killed (as above) and murine- (spleens, livers, intestines, intestinal fecal pellets) and (where present; i.e., the infected animals) parasite- material (worms, liver eggs, and intestinal eggs) obtained. These materials were processed for downstream parasitological, immunological, histological, pathological, transcriptional, and microbiome analyses.

### Phase 2 PK Studies.

Five uninfected female mice (2 × 8HUM and 3 × WT) were administered a single oral dose of rac-PZQ (25 mg/kg for 8HUM and 400 mg/kg for WT) as a fine suspension in 0.5% (w/v) hydroxypropyl methylcellulose (HPMC) at a dose volume of 5 mL/kg. Dose formulations were prepared on the day of dosing. Serial blood samples (10 μL per sample) were collected from the lateral tail vein prior to dosing, then at 5, 15, 30, 60, 120, 240, 360, and 480 min after administration. Each blood sample was diluted into 90 μL of Milli-Q ultrapure water and stored at −20 °C prior to bioanalysis by U(H)PLC-MS/MS.

### Phase 3 Combined PK/Efficacy Studies.

All mice included in the study were housed in Aberystwyth University’s biological research facility for 4 wk before investigations began. For combined PK/efficacy studies, 8HUM and WT mice (6- to 8-wk-old females) were percutaneously exposed to 120 cercariae/mouse. At 41 d post infection, groups of animals (9 to 10 animals per group) were administered, via oral gavage, a single dose (100 mg/kg and 200 mg/kg for 8HUM, 400 mg/kg for WT) of rac-PZQ as a fine suspension in 0.5% (w/v) HPMC at a dose volume of 5 mL/kg. Three animals from each dosing group were selected at random for PK evaluation and serial blood sampling of these animals was performed as outlined above. Infected animals (9 to 10 animals per group; both 8HUM and WT) treated with HPMC (5 mL/kg) only were included as negative controls for the efficacy experiment. At 47 d postinfection, animals were killed (as above) and parasite material obtained and processed as before.

## Supplementary Material

Appendix 01 (PDF)

Dataset S01 (XLSX)

Dataset S02 (XLSX)

Dataset S03 (XLSX)

Dataset S04 (XLSX)

Dataset S05 (XLSX)

Dataset S06 (XLSX)

Dataset S07 (XLSX)

Dataset S08 (XLSX)

Dataset S09 (XLSX)

## Data Availability

Transcriptome and metagenome data have been deposited in ENA (PRJEB94950) (44). All other data are included in the manuscript and/or supporting information.
