# Supplementary material for: Use of a cytochrome P450 humanized mouse model to refine schistosomiasis drug discovery
Source: Proc Natl Acad Sci U S A. 2026 Apr 10;123(15):e2600197123. doi: 10.1073/pnas.2600197123 (PMC13079396; doi:10.1073/pnas.2600197123)
Supplement: Supplementary file 1 — Appendix 01 (PDF) [file pnas.2600197123.sapp.pdf]

Supplementary Information Appendix

Use of a cytochrome P450 humanised mouse model to refine schistosomiasis drug discovery.

Authors: Sarah D. Davey[1†], Josephine E. Forde-Thomas[1†], Benjamin J. Hulme[1†], Kristin Lees[1†], Alice H. Costain[2], Mary Evans[1], Gabriel Rinaldi[1‡], Laura Frame[3], Laste Stojanovski[3], Frederick R. C. Simeons[3], Amy Tavendale[3], A. Kenneth MacLeod[3], Remi Pichon[1‡], Yi-Hsuan Lee[1,4], Oktawia Polak[1§], Iain W. Chalmers[1], Bismark Dankwa[1||], Brenda Kisia Odhiambo[1], Victor Hugo Guimaraes[5], Matthew Hegarty[1], Martin T. Swain[1], Wayne Aubrey[4], Nicola Caldwell[3], Andrew S. MacDonald[2¶], Ian H. Gilbert[3], Beatriz Baragaña[3], Kevin D. Read[3] and Karl F. Hoffmann[1*]

Affiliations: [1]Department of Life Sciences; Aberystwyth University, Aberystwyth, Wales, SY23 3DA, UK. [2]Lydia Becker Institute of Immunology and Inflammation, Division of Immunology, Immunity to Infection & Respiratory Medicine, School of Biological Sciences; The University of Manchester, Manchester, England, M13 9NT, UK. [3]Drug Discovery Unit, Division of Biological Chemistry and Drug Discovery, School of Life Sciences; University of Dundee, Dundee, Scotland, DD1 5EH, UK. [4]Department of Computer Science; Aberystwyth University, Aberystwyth, Wales, SY23 3DA, UK. [5]Laboratory of Inflammation and Infectious Diseases, Department of Morphology and Pathology; Federal University of São Carlos, Brazil, CEP 13565-905.

[†]These authors contributed equally to this work
[‡]Current address - Department of Biology; University of Oxford, Oxford, England, OX1 3EL, UK.
[§]Current address - Countermeasure Development, Evaluation & Preparedness, Vaccine Development and Evaluation Centre; UK Health Security Agency, Salisbury, England, SP4 0JG, UK.
[||]Current address - Centre for Medicines Discovery; University of Oxford, Oxford, England, OX3 7FZ, UK.
[¶]Current address - Institute of Immunology and Infection Research, School of Biological Sciences; The University of Edinburgh, Edinburgh, Scotland, EH9 3JT, UK.
*To whom correspondence should be addressed; krh@aber.ac.uk

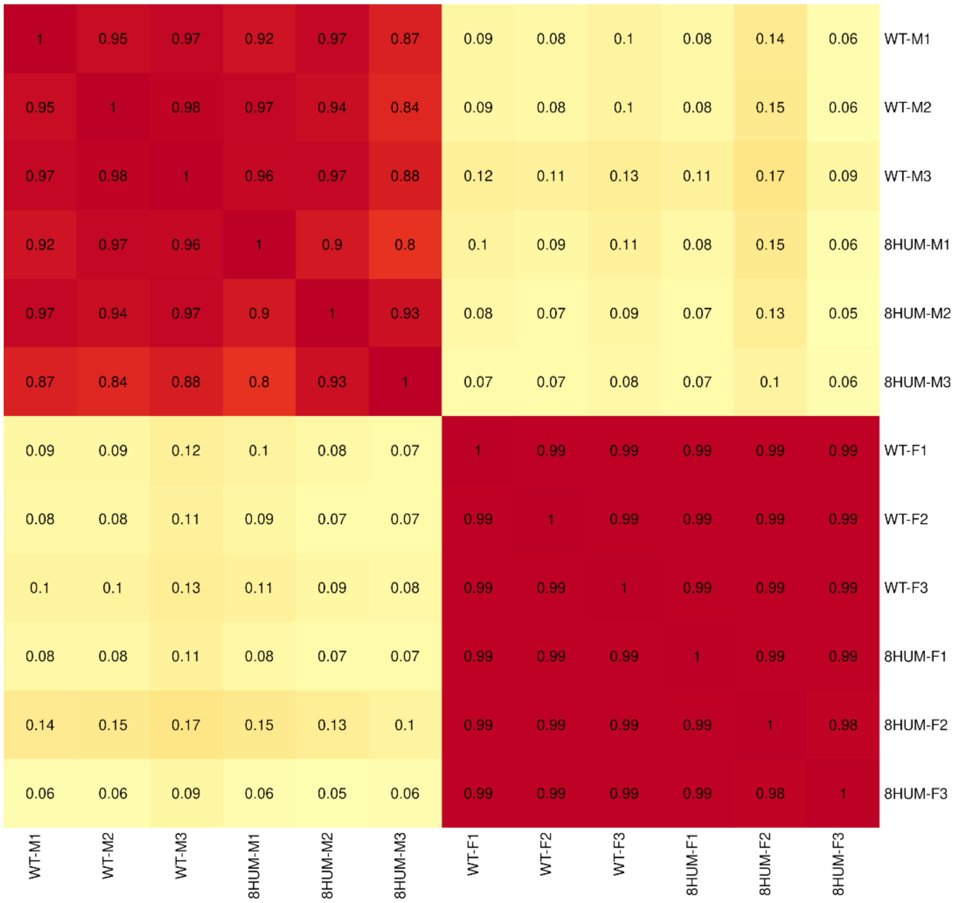

Fig. S1. Pearson correlation matrix of transcript expression between samples for male (n = 3 per strain) and female (n = 3 per strain) worms derived from wild-type (WT) and 8HUM mice.

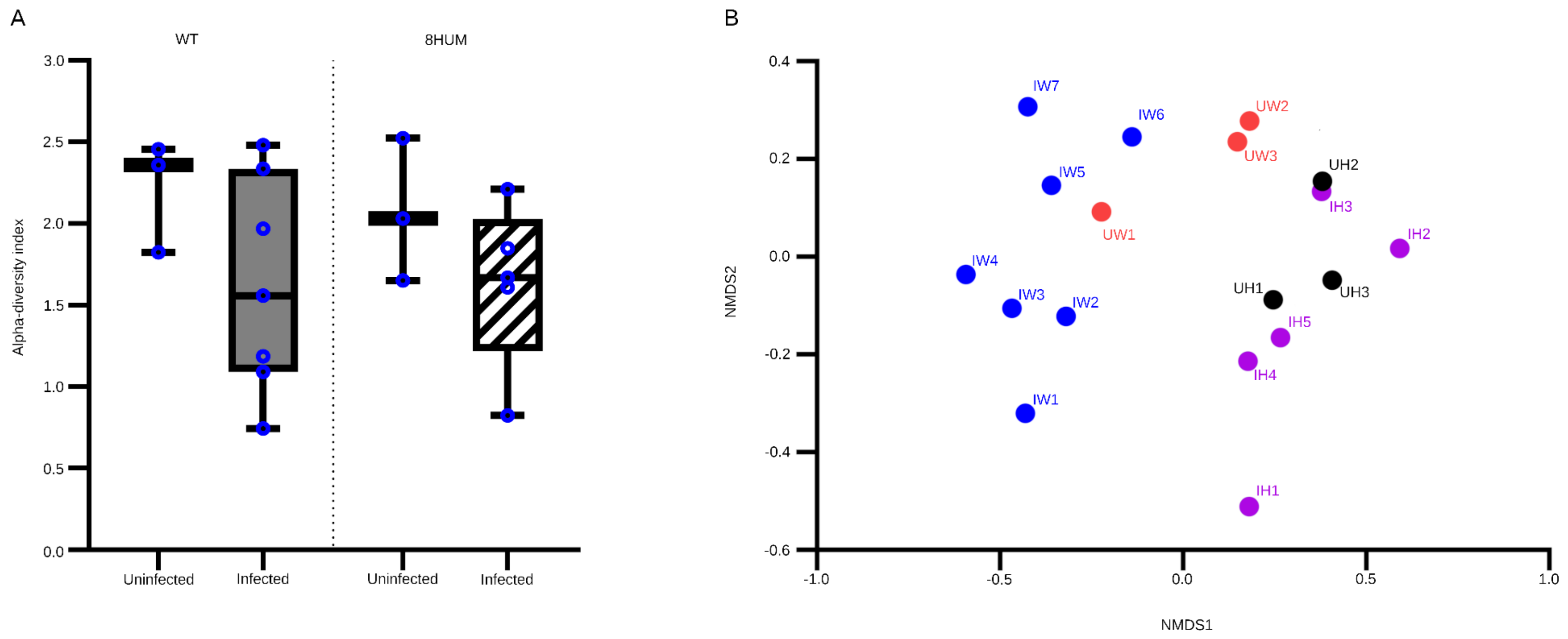

Fig. S2. Compositional profiles of the faecal microbiomes for each mouse. **(A)** Alpha diversity using species richness and Shannon index (*p* value: 0.31284; Kruskal–Wallis statistic: 3.5617). **(B)** Beta diversity using non-metric multidimensional scaling (NMDS) (PERMANOVA: F-value: 1.9469; R^2: 0.29438; *p* value: 0.112; NMDS Stress = 0.12384). WT uninfected: UW1-3 (red), WT infected: IW1-7 (blue), 8HUM uninfected: UH1-3 (black), and 8HUM infected: IH1-5 (purple).

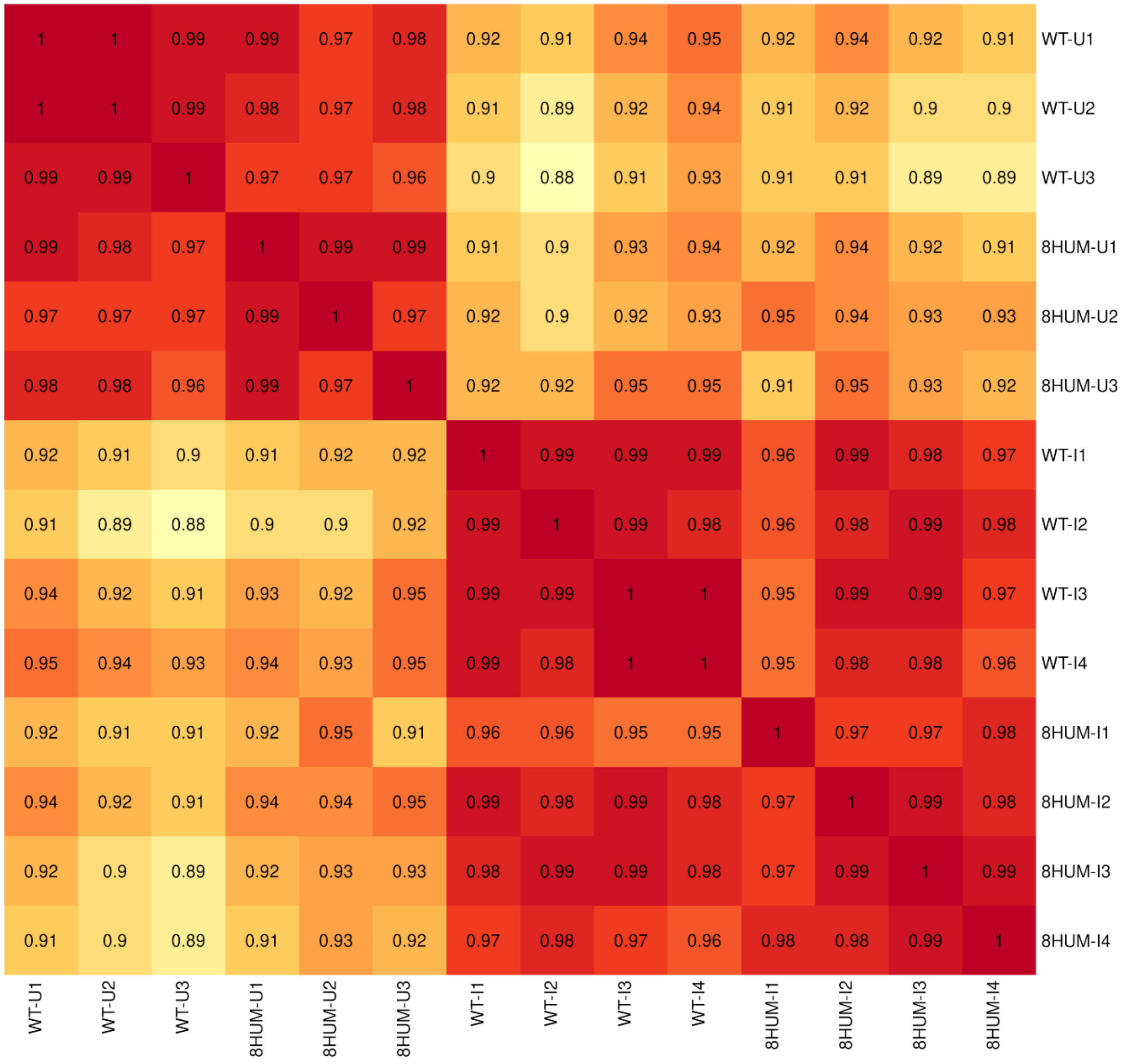

Fig. S3. Pearson correlation matrix of gene expression between liver samples for uninfected (n = 3 per strain) and *S. mansoni* infected (n = 4 per strain) wild-type (WT) and 8HUM mice.

A

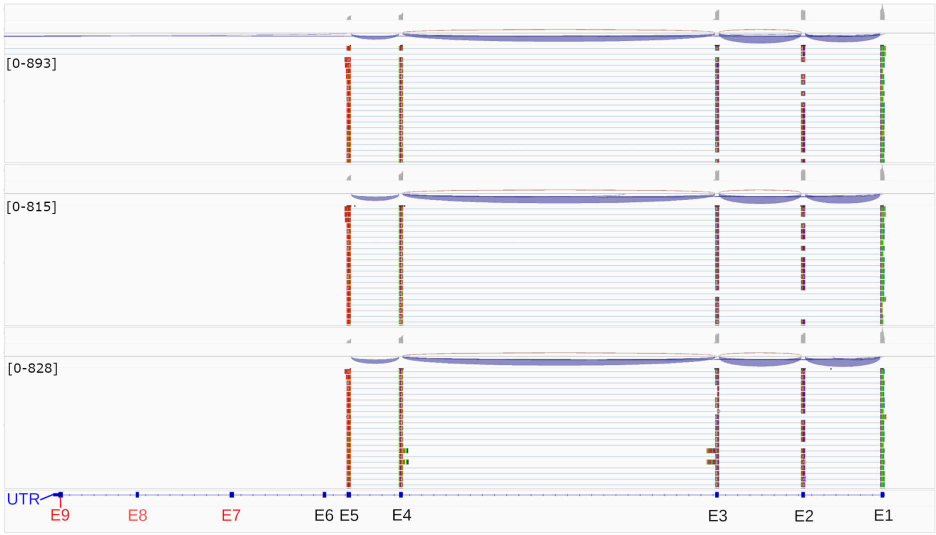

Cyp2c70, mouse residual

B

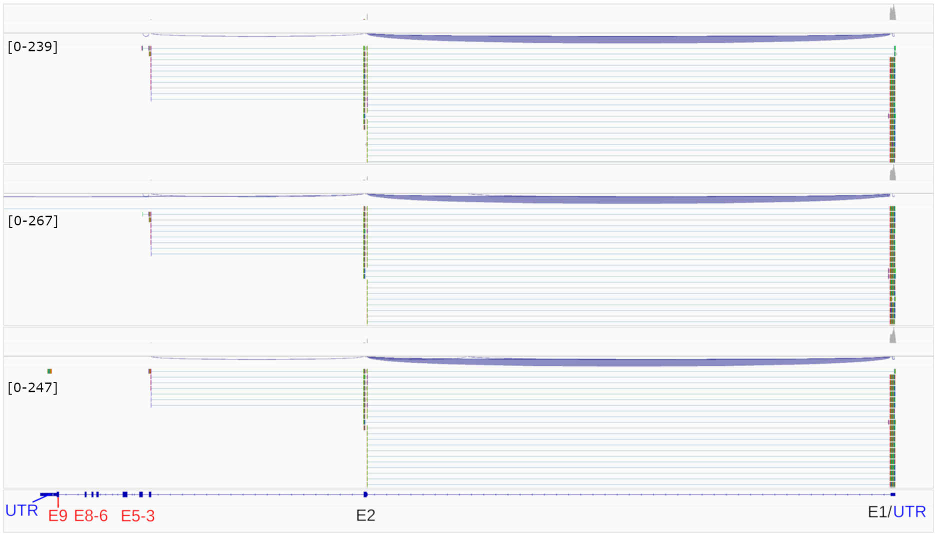

PXR, mouse residual

C

Mouse gene	Maximum expression, 8HUM-U/I (TPM)	Source of residual reads
CAR	18.85	Mostly UTR, very low number of poor coverage reads in exons 2, 3 and 4
Cyp1a2	11.96	100% UTR
Cyp2c70	121.04	Exons 1-5 only, no reads aligning to exons 6-9 (see **A**.)
Cyp3a57	13.41	100% UTR
PXR	36.67	UTR < exon 1, v. low number poor coverage reads exons 2 & 3, some in UTR > exon 8 (see **B.**)

Fig. S4. IGV interrogation of residual mouse reads mapping to 8HUM samples following removal of reads mapping to humanised genes (panels A and B). Where mouse genes were detectable in 8HUM samples at >10 TPM, explanations for residual reads are provided in Table C.

Supplemental methods

Microsomal incubations

Microsome preparations and microsomal incubation procedures were described previously (1). Briefly, rac-PZQ was added at a final concentration of 0.5 µM to microsomes in buffer (0.5 mg/mL protein in 50 mM potassium phosphate, pH 7.4), and reactions initiated with addition of excess NADPH (final concentration 0.8 mg/mL). Aliquots of 50 µL were taken at 0, 3, 6, 9, 15 and 30 min, immediately mixed with two volumes of acetonitrile containing internal standard (IS, sulfadimethoxine, 50 ng/mL) and kept on ice. When all samples were collected, 250 µL of 20% acetonitrile was added to each and these were centrifuged for 10 min at 3,000 × *g* at ambient temperature. Supernatants were analysed by LC-MS/MS immediately.

LC-MS/MS analysis

PK samples were analysed on an Acquity UPLC system coupled to a Xevo TQ-XS, operated using MassLynx software version 4.2 (Waters, Wilmslow, UK). Chromatographic separation was achieved using a previously described method (2) with minor modifications. Briefly, a Lux Cellulose-2 column (150 × 4.6 mm; particle size 3 µm, Phenomenex, UK), with a SecurityGuard Lux Cellulose-2 (4 x 3.0 mm ID) held at 25 °C, was used. The flow rate was 1.2 mL/min. The elution gradient was: 0.0 – 1.0 min: 70% B, 1.0 – 2.0 min: 70 to 90% B, 2.0 to 9.5 min: 90% B, 9.5 – 10.0 min: 95 – 0% B, 10.0 – 10.5 min: 0 – 70% B and 10.5 – 11.5 min: 70% B. Mobile phase A was 20 mM ammonium formate in LC-MS grade water and mobile phase B was acetonitrile. The analytes were detected by multiple reaction monitoring (MRM) in positive electrospray mode. MRM transitions were 313.2 > 203.1 for PZQ and 329.1 > 203.1 for 4OH-PZQ. Mass spectrometer parameters were as follows: capillary voltage of 0.8 kV, desolvation temperature of 600 °C, desolvation gas flow of 1000 L/h, cone gas flow of 150 L/h and source temperature of 150 °C.

Where samples containing rac-PZQ were analysed, only the *R* enantiomer (first of two peaks) was quantified. For 4OH-PZQ, two peaks were also observed due to the chiral chromatographic method used. Although it was considered likely that the first peak conformed to the *R* enantiomer and the second peak conformed to the *S* enantiomer (similar to what was observed for rac-PZQ), this could not be confirmed in the absence of authentic standards. Therefore, integrated areas of both peaks were summarised for reporting the total 4OH-PZQ levels.

Microsomal incubation samples were analysed on an Acquity UPLC system coupled to a Xevo TQS-Micro. Chromatographic separation was achieved using an Acquity UPLC BEH C18 column, 50 × 2.1 mm, particle size 1.7 µm (Waters, part number 186002350) held at 45 °C, with mobile phase A of 0.01% formic acid in Milli-Q water, and mobile phase B of 0.01% formic acid in LC-MS grade methanol. The gradient programme was as follows: 0.0 – 0.3 min: 5% B, 0.3 – 1.3 min: 5 to 95% B, 1.3 – 1.8 min: 95% B and 1.8 – 1.81 min: 95 – 5% B. Mass spectrometer parameters were as for PK sample analysis, with the exception of desolvation temperature which was set to 500 °C and cone gas flow which was 15 L/h. *R*- and *S*- enantiomers were not separated using this methodology and results are, therefore, reported as intrinsic clearance of racemic praziquantel.

Intrinsic clearance determination

Intrinsic clearance (CLint) was calculated as previously described (1). Briefly, following LC-MS/MS analysis, the exponential decay rate constant (*k*) of the compound under study was calculated using XLfit (IDBS, Woking, UK) and multiplied by the incubation volume and microsomal protein concentration. Data were scaled to mL/min/g (liver) using the scaling factors

of 48 and 39.7 mg microsomal protein per g of liver for mouse and human, respectively. Lower and uppers limits of quantitation were 0.011 and 0.1 mL/min/mg, respectively.

Quantitative bioanalysis of PK samples

Drug and metabolite levels were quantified as previously described (1). Briefly, PK samples were extracted into three volumes of acetonitrile containing internal standard (IS, sulfadimethoxine at 100 ng/mL). Calibration standards (CS) and quality controls (QC) – prepared by spiking of compounds into diluted blank blood – were extracted in parallel. Quantification was achieved by interpolation of sample response onto CS response using TargetLynx (Waters). Analytical acceptance criteria were reported previously (1). PK parameters were determined by non-compartmental analysis in Phoenix WinNonlin (Certara, Sheffield, UK).

Parasitology

Schistosomes were manually counted using a camera mounted to a Nexius Zoom dissection microscope (Euromex). Fresh livers and intestines (after removal of faecal pellets from the colon and flushing residual material with 10 mL phosphate buffered saline, PBS), were weighed and frozen at -80 ^{o}C until downstream processing. Faecal pellets were also stored at -80 ^{o}C until use. Livers and intestines were thawed and digested in 10 mL/g tissue 4% w/v potassium hydroxide (KOH) in ddH_2O for 16 h at 37°C with agitation as previously described (3). Eggs liberated from tissues were washed five times with 50 mL 0.01 M PBS, passed through a 100 µm cell strainer to remove residual debris and resuspended in 5 mL 4% (v/v) formaldehyde in ddH_2O. Three x 10 µL aliquots per sample were diluted in water to a final volume of 200 µL, placed in a 96-well dark walled plate and imaged at 2.5x magnification using a Cytation 7 multi-modal imager (Agilent)

with the GFP filter. Eggs were manually counted and eggs per gram of tissue (EPG) was calculated by extrapolating the mean number of eggs across three aliquots to the total volume of original sample and dividing by the tissue weight.

Splenocyte culture and cytokine assays

Spleens were removed from each mouse, prior to reverse perfusion of the hepatic-portal venous system, and individual single cell suspensions prepared. Splenocytes were plated in 24-well tissue culture plates at a final concentration of 4 x 10^6 cells/mL in complete media and cultured for 72 h in a humidified atmosphere containing 5% CO_2 as previously described (4). Cells were stimulated with ConA (5 μg/mL), SEA (20 μg/mL), SWAP (50 μg/mL) or medium alone. SEA and SWAP were generated as described previously (5, 6) using mixed-sex adult worms as starting material (for SWAP). Supernatants were harvested at 72 h and assayed for IL-4, IL-5, IL-10, IFN-gamma, IL-13 and IL-17 by sandwich ELISA as previously described (7).

Measurement of SWAP- and SEA-specific Ab responses

For assessment of anti-SEA and anti-SWAP Ig levels, serum samples prepared from whole blood collected at 46 days post-infection were assayed from individual animals as previously described with slight modifications (4). Briefly, individual mouse serum was serially diluted 1/100 - 1/102, 500 in 1% w/v BSA in PBS/0.05% Tween-20 and 50 μL was added to appropriate wells. Fifty μL of isotype specific horse-radish peroxidase (HRP) conjugated rabbit anti-mouse Abs (IgG_1 = PA186329, Invitrogen; IgG_{2b} = 43R-IR032hrp_1MG, Fitzgerald Industries; total IgG = 616520, Invitrogen) in 1% w/v BSA in PBS/0.05% Tween-20 diluted at 1/1000 (measurement of IgG_1 and IgG_{2b}) or 1/2000 (measurement of total IgG) were added to the wells and incubated at 37 °C for 2 h. Reactions were

developed at room temperature (RT) until the desired signal was reached (IgG_1 = 14 – 21.5 min, IgG_{2b} = 22 – 36 min and Total IgG = 7.5 – 10.5 min), terminated with 100 µL of 1% w/v SDS and the OD (absorbance) at 405 nm was determined using a PolarStar Omega microtiter plate reader (BMG Labtech). Specific SWAP- and SEA-isotype titres were represented by the product of absorbance of a single point on the linear portion of the dilution curve.

Liver histology

At day 47 post-infection, after reverse perfusion of the hepatic-portal venous system, approximately half of each liver was removed from individual mice, fixed in 10% buffered formalin for 24 h and then transferred to 70 % ethanol. Fixed liver samples were embedded in paraffin and 5 µm sections prepared for staining with haematoxylin and eosin (H&E) as well as picrosirius red (PSR) (8). To estimate percentage collagen within granuloma boundaries, PSR stained granulomas (counter stained with fast green for contrast) from 10x magnification micrographs captured using a Pannoramic 250 slide scanner were exported using 3D HISTECH Pannoramic Viewer (version 1.15.4). Granuloma boundaries were masked manually using the CVAT annotation service (www.cvat.ai). Annotations were exported in COCO 1.0 format (9), from which total areas (μm^2), extrapolated spherical volumes (μm^3) and percentage PSR stain (as a proxy for collagen) were calculated within object masks using a custom Python script (v. 3.10.12, available via public repository https://github.com/flukebio97/8HUM_mouse/).

Microbiome analyses of faecal samples

Genomic DNA was extracted from colonic content samples (day 47 post-infected- and uninfected mice) as well as no-template negative controls, using the PowerSoil DNA Isolation Kit (QIAGEN)

following the manufacturer's protocol. DNA library preparation and sequencing were performed using the Illumina MiSeq platform on the V3-V4 region with paired-end 250 bp reads. Raw 16S rRNA amplicon sequencing data are available from the European Nucleotide Archive (ENA) database under BioProject PRJEB94950 (10).

All 16S rRNA sequencing datasets were initially processed using Cutadapt (version 4.1). Amplicon sequence analysis was conducted using the DADA2 pipeline (version 1.16). Quality filtering was performed with the filterAndTrim function, applying the following parameters: maxN = 0, maxEE = c(2, 2), truncQ = 2, rm.phix = TRUE, and truncLen = c(220, 200). Chimeric amplicon sequence variants (ASVs) were removed using the removeBimeraDenovo function with the 'consensus' method. Taxonomic assignment was performed by aligning ASVs to the SILVA database (version 138) curated for DADA2.

The resulting ASV and taxonomy tables were further analysed using MicrobiomeAnalyst (11). Data were normalised by cumulative sum scaling (CSS) and rarefied to a uniform sequencing depth of default reads per sample. Alpha diversity metrics, including species richness and Shannon diversity index, were compared between groups using unpaired and pairwise Kruskal-Wallis tests. Beta diversity was evaluated using nonmetric multidimensional scaling (NMDS) based on Bray–Curtis dissimilarity metrics. Statistical differences between groups were assessed by Permutational Multivariate Analysis of Variance (PERMANOVA).

RNA-sequencing (RNA-Seq)

At 47 days post-infection, 250 mg of liver tissue/mouse was removed from the left lobe prior to reverse perfusion of the hepatic-portal venous system and total RNA isolated as previously described (12). Total RNA was also isolated from adult male and female worms at 47 days post-

infection. Here, schistosomes from three individual mice (WT, n = 3 and 8HUM, n = 3) were pooled by sex and RNA isolated using the MasterPure Complete DNA and RNA Purification Kit (Cambio) following the manufacturer's instructions but with a 60-minute DNase treatment. The quality and quantity of murine and schistosome total RNA samples were assessed via analysis on a ThermoFisher Nanodrop 2000 and an Agilent Bioanalyzer 2100. Total RNA samples were submitted to BGI Tech Solutions Co., Limited (Poland) for library construction and DNBSEQ eukaryotic strand-specific transcriptome resequencing at paired-end 100 bp read length with ≥22 million clean reads per sample. All RNA-Seq samples used in this study are available to download from ENA BioProject PRJEB94950 (10).

Transcriptomic assembly and analysis

All mouse samples were assembled against the *Mus musculus* reference genome (GRCm39, NCBI RefSeq accession GCF_000001635.27) by BGI Tech Solutions Co., Limited. Briefly, reads were mapped using HISAT (v.2.04) (13) followed by quantification of transcript expression using Bowtie2 (v.2.25) (14) and RSEM (v.1.2.8). To delineate human sequence reads from mouse reads for the replaced cytochromes and transcription factors, a chimeric HISAT2 reference was built to include both human and mouse cytochrome sequences in the GRCm39 core transcriptome. All samples (WT and 8HUM) were remapped against this reference for the purposes of cytochrome characterisation only (i.e., not for downstream analyses subsequently described). This index is publicly available via the Zendo digital archive (15). Differential expression was quantified using DESeq2 (16) with a minimal q-value cutoff of ≤ 0.05. Functional annotation of genes was performed for GO terms by mapping genes to the Gene Ontology database

(http://www.geneontology.org/) followed by Gene Set Enrichment Analysis (GSEA) in the phyper R package.

Given that *S. mansoni* is a non-reference species, assembly and analysis was performed in-house at AU. Using version 10 (v.10) of the *S. mansoni* genome assembly on WormBase ParaSite (PRJE36577), a genome index was constructed using soft-masked sequences and mRNA transcript decoys in Salmon (k-mer length = 27) with collapsing of identical transcripts permitted (indexing transcripts for 9, 980 protein coding genes). Transcript quantification was also performed using Salmon, followed by differential expression analysis by edgeR (17) in Trinity (v.2.15.2) with cut-offs set at $q \leq 0.001$ and a minimum absolute $\log_2$FC of 2 (4-fold change).

Whole transcriptome heatmaps were generated using the heatmap.2 function of Bioconductor's gplots with the RcolorBrewer palette library in R (v. 4.5.1) using Rstudio (v. 2024.12.0). Default parameters were used according to documentation, with row-means dendrogram clustering applied using Rowv = TRUE. Subset heatmaps and bubbleplots for GSEA analysis were generated in Python (v.3.10.12) by leveraging matplotlib in a custom script.

Statistical analyses

Distributional assessments were performed prior to all statistical testing using GraphPad Prism (v.8.4.3). Parametric datasets were compared by either a one-way or two-way ANOVA with Tukey's Multiple Comparisons Test. For non-parametric data, differences were assessed using a Mann-Whitney U test for two groups. All significance values given in this manuscript are adjusted as required and significant p or q values are abbreviated as *, **, *** and **** for < 0.05, < 0.01, < 0.001 and < 0.0001, respectively. All statistical comparisons and corrections for transcriptomic

data were performed using default options within the respective differential expression software (edgeR for worm data and DESeq2 for mouse data).

References

1. A. K. MacLeod *et al.*, Acceleration of infectious disease drug discovery and development using a humanized model of drug metabolism. *Proc Natl Acad Sci U S A* **121**, e2315069121 (2024).
2. I. Meister *et al.*, Development and validation of an enantioselective LC-MS/MS method for the analysis of the anthelmintic drug praziquantel and its main metabolite in human plasma, blood and dried blood spots. *J Pharm Biomed Anal* **118**, 81-88 (2016).
3. A. W. Cheever, Conditions affecting the accuracy of potassium hydroxide digestion techniques for counting Schistosoma mansoni eggs in tissues. *Bull World Health Organ* **39**, 328-331 (1968).
4. K. F. Hoffmann, S. L. James, A. W. Cheever, T. A. Wynn, Studies with double cytokine-deficient mice reveal that highly polarized Th1- and Th2-type cytokine and antibody responses contribute equally to vaccine-induced immunity to Schistosoma mansoni. *J Immunol* **163**, 927-938. (1999).
5. E. J. Pearce *et al.*, Schistosoma mansoni in IL-4-deficient mice. *Int Immunol* **8**, 435-444 (1996).
6. A. T. Vella, E. J. Pearce, CD4^{+} Th2 response induced by *Schistosoma mansoni* eggs develops rapidly, through an early, transient, Th0-like stage. *J.Immunol.* **148**, 2283-2288 (1992).
7. A. H. Costain *et al.*, Dynamics of Host Immune Response Development During Schistosoma mansoni Infection. *Front Immunol* **13**, 906338 (2022).
8. K. Su *et al.*, SOX9 plays an essential role in myofibroblast driven hepatic granuloma integrity and parenchymal repair during schistosomiasis-induced liver damage. *PLoS Pathog* **21**, e1012928 (2025).
9. T.-Y. Lin *et al.* (2014) Microsoft coco: Common objects in context. in *Computer Vision–ECCV 2014: 13th European Conference, Zurich, Switzerland, September 6-12, 2014, Proceedings, Part V 13* (Springer), pp 740-755.
10. S. D. Davey *et al.*, Use of a cytochrome P450 humanised mouse model to refine schistosomiasis drug discovery. https://www.ebi.ac.uk/ena/browser/view/PRJEB94950. Deposited 31-10-2025.
11. A. Dhariwal *et al.*, MicrobiomeAnalyst: a web-based tool for comprehensive statistical, visual and meta-analysis of microbiome data. *Nucleic Acids Res* **45**, W180-W188 (2017).
12. K. F. Hoffmann, J. M. Fitzpatrick, Gene expression studies using self-fabricated parasite cDNA microarrays. *Methods Mol Biol* **270**, 219-236 (2004).
13. D. Kim, B. Langmead, S. L. Salzberg, HISAT: a fast spliced aligner with low memory requirements. *Nat Methods* **12**, 357-360 (2015).
14. B. Langmead, S. L. Salzberg, Fast gapped-read alignment with Bowtie 2. *Nat Methods* **9**, 357-359 (2012).

15. S. D. Davey, K. F. Hoffmann, Custom HISAT index for 8HUM mouse model [Data set]. In PNAS (1.0).
16. M. I. Love, W. Huber, S. Anders, Moderated estimation of fold change and dispersion for RNA-seq data with DESeq2. *Genome biology* **15**, 550 (2014).
17. M. D. Robinson, D. J. McCarthy, G. K. Smyth, edgeR: a Bioconductor package for differential expression analysis of digital gene expression data. *Bioinformatics* **26**, 139-140 (2010).
